# Household Determinants of Liquified Petroleum Gas (LPG) as a Cooking Fuel in SW Cameroon

**DOI:** 10.1007/s10393-018-1367-9

**Published:** 2018-10-01

**Authors:** Daniel Pope, Nigel Bruce, James Higgerson, Lirije Hyseni, Debbi Stanistreet, Bertrand MBatchou, Elisa Puzzolo

**Affiliations:** 1 Department of Public Health and Policy, University of Liverpool, Whelan Building, Quadrangle, Liverpool, L69 3GB UK; 2 Douala General Hospital, Douala, Cameroon

**Keywords:** Household wealth, Socio-economic status, Clean fuel, Adoption, Household air pollution

## Abstract

Currently 70% of the population in Cameroon are reliant on solid fuel for cooking (90% in rural communities) and the associated household air pollution contributes to significant mortality and morbidity in the country. To address the problems of energy security, deforestation and pollution the government has developed a strategy (Masterplan) to increase use of liquified petroleum gas (LPG) as a cooking fuel from 12% to 58% by 2030. As a clean fuel scaled adoption of LPG has the potential to make significant positive impacts on population health. The LPG Adoption in Cameroon Evaluation (LACE) studies are assessing in the community (i) barriers and enablers for and (ii) local interventions to support, adoption and sustained use of LPG. A census survey conducted for LACE in rural and peri-urban regions of SW Cameroon provided an opportunity to investigate current fuel use patterns and factors associated with primary and exclusive use of LPG. A cross-sectional survey of 1577 households (1334 peri-urban and 243 rural) was conducted in March 2016 using standardised fuel use and household socio-demographic questions, administered by trained fieldworkers. Wood (40.7%) and LPG (51.1%) were the most frequently reported fuels, although the dominant fuels in rural and peri-urban communities were wood (81%) and LPG (58%) respectively. Fuel stacking was observed for the majority of LPG using households (91% of peri-urban and 99% of rural households). In rural homes, a higher level of education, access to sanitation and piped water and household wealth (income and asset ownership) were all significantly associated with LPG use (*p* < 0.05). In peri-urban homes, younger age, access to sanitation and piped water and increasing education were significantly associated with both any and exclusive use of LPG (*p* < 0.05). However, whilst household wealth was related to any LPG use, there was no relationship with exclusive use. Results from this census survey of a relatively well-established LPG market with lower levels of poverty and high levels of education than Cameroon as a whole, find LPG usage well below target levels set by the Cameroon government (58% by 2030). Fuel stacking is an issue for the majority of LPG using households. Whilst, as observed here, education, household wealth and socio-economic status are well recognised predictors of adoption and sustained use of clean modern fuels, it is important to consider factors across the whole LPG eco-system when developing policies to support their scaled expansion. A comprehensive approach is therefore required to ensure implementation of the Cameroon LPG Masterplan achieves its aspirational adoption target within its stated timeframe.

## Introduction

Approximately 700 million people are dependent on the use of solid fuel (predominantly wood) for cooking in sub-Saharan Africa, a figure that continues to rise despite decreases in other regions. It is estimated that almost 900 million people will be reliant on biomass fuel by 2020 as efforts to expand access to modern cleaner energy (electricity and gas) are far outpaced by population growth (Lambe et al. 2015). The negative impacts of traditional solid fuel reliance are well established (Smith et al. 2014). Unsustainable harvesting of fuel wood and associated deforestation are a major issue in many lower- and middle-income countries (LMIC) (Subedi et al. 2014) with 70% of deforestation in Africa attributed to wood fuel demand and predicted to increase up to 83% by 2030. The negative impacts on climate and health from household air pollution (HAP) are also well established with HAP estimated to contribute 25% of global black carbon, the most important influence on climate change after CO_2_ (Bailis et al. 2005). In 2016, in sub-Saharan Africa, exposure to HAP from cooking with solid fuel was estimated to result in 520,000 premature deaths (6.8% of all deaths) and almost 23 million disability-adjusted life years (DALYS; 4.6% of total), from ischaemic heart disease, stroke, lung cancer and chronic obstructive pulmonary disease in adults and pneumonia in children (IHME 2016). The economic costs from solid fuel reliance in sub-Saharan Africa are also substantive with estimates of approximately US$37 billion per year (2.8% of GDP), largely resulting from lost productivity from the necessity to gather and cook with the fuels (approximately US$30 billion) (Lambe et al. 2015). The associated health and economic burdens are largely born by women and girls due to traditional gender-based roles around cooking, making reliance on solid fuels a major source of gender inequality (Austin and Mejia 2017).

In 2014, the WHO published indoor air quality guidelines on household fuel combustion to address what they called ‘the greatest environmental health risk in the world today’ (WHO 2014). The guidelines were developed for public health policy makers and stakeholders in the energy sector to understand the best approaches for reducing HAP. One key recommendation from the guidelines was that clean fuels should be prioritised to meet target emission levels to protect health and that ‘governments and their implementing partners should develop strategies to accelerate efforts to meet (these emission rates)’. Making clean energy solutions (including gas, electricity and biogas) more widely available, especially among the world’s poorest people, is also a necessity to achieve Sustainable Development Goal 7 to ‘ensure universal access to affordable, reliable, sustainable and modern energy for all’ by 2030 (WHO 2016).

Liquified petroleum gas (LPG) is widely available across geographical regions of sub-Saharan Africa, although with limited use in many countries, and is an efficient and safe cooking fuel with the potential to deliver benefits for health, climate, environment and development (Bruce et al. 2017). Accordingly, a number of sub-Saharan governments including Ghana, Kenya and Cameroon have made it a priority to provide a majority of their populations with LPG for reasons such as addressing air pollution, forest preservation and economic development (Van Leeuwen et al. 2017, Bruce et al. 2017). In Cameroon, where approximately 70% of the population use solid fuel for cooking (more than 90% in rural communities) (DHS 2011), the government has a target to increase LPG adoption from approximately 20% of the population to 58% (18 million people) by 2030 to address problems of energy security, deforestation and pollution. To this end, with support from the Global LPG Partnership, they have developed a master plan to inform strategies to scale adoption through regulation, infrastructure, supply and access, published in 2016 (SE4All 2017).

One important aspect in facilitating the widespread transition from solid fuel to LPG for cooking is to understand how best to encourage and support households to both adopt LPG and use it exclusively in a sustained way. The LPG Adoption in Cameroon Evaluation (LACE) studies was launched in 2016 by the University of Liverpool, UK, to (1) identify potential enablers and barriers for adoption and sustained use of LPG and (2) test interventions to support communities in making the transition to cleaner fuel. The studies were conducted after publication of, but independent from, the LPG Masterplan to provide evidence to support scaled transition from solid fuel to LPG from a community perspective. A first step of the LACE studies was to identify household patterns of fuel use in the community and the factors that influence these choices through population surveys. This paper reports on findings from the LACE population surveys in south-west Cameroon describing patterns of fuel use and cooking practices in rural and peri-urban communities and demographic and socio-economic characteristics associated with the use of solid fuel and LPG.

## Methods

Cross-sectional population-based surveys were conducted to obtain relevant information on household characteristics and fuel use patterns. Communities were selected from the two main districts of south-west Cameroon (an Anglophone coastal region with a relatively well-established LPG market and infrastructure): Limbe, comprising peri-urban settings, and Buea located approximately 15 km from Limbe with distinct rural communities. All households from rural Buea (approximately 500 from Boana, Bojoke and Upper/Middle/Lower Bojongo villages) were eligible for the survey. For peri-urban Limbe (Mile 4, Middle Farm and Bota), the population was approximately 20,000–30,000 households and a stratified random sampling approach was adopted to represent the geographical areas of the communities. Using a demographic health map, available from the Mile 4 health administrative building, indicating household locations according to designated ‘quarters’ and the approximate size of the population for each quarter (number of houses and size of the houses), a total sample of 1600 households was selected.

In terms of access to LPG as a household cooking fuel, for the peri-urban communities, LPG cylinders are widely available in Limbe which has good market penetration from a number of the Cameroon marketing companies (the leading ones in the community being Tradex and GlocalGaz). Households can exchange cylinders from a range of brands from small retail outlets located within the peri-urban communities or from large retailers on the main roads through the town. (These retailers are general stores that sell cylinders in addition to other merchandise.) It is also possible to exchange cylinders at one of the petrol stations in Limbe. Due to a lack of retail outlets located in the rural communities, households typically have to travel to Limbe (up to 10 km) to purchase LPG. They also have cylinders delivered (by taxi or motorbike), but this incurs an additional delivery charge.

The surveys were primarily designed to provide a sampling frame for the LACE studies and so included questions on the demographic makeup of homes that could be used for sampling including household composition (residents including children), socio-economic status (education, sanitation and water access), income (asked as a closed question for participants to check an income bracket for their monthly household income after piloting found this was preferable to respondents compared to an open question), assets (originally including ten items but restricted to five after some were identified as not relevant to the study population or did not vary by household) and current primary and secondary fuel use (for cooking, lighting and heating). All survey questions were based on those previously used in field-based research conducted by Practical Action in Nepal, Kenya and Sudan (PAC 2005). Questions on fuel use were based on WHO household energy survey questionnaire designed to monitor Sustainable Development Goal 7.1.2 on primary reliance on clean fuels and technologies (SDG 2017). The survey questionnaire was extended to include specific questions on (1) patterns of cooking (including seasonality) and foods cooked, (2) preference questions on perspectives of attributes of LPG, (3) details of how LPG is used in households that have adopted LPG and (4) details of future potential use of LPG in current non-using households.

The surveys were conducted using personal digital assistants (PDAs) over a 2-week period in March 2016 by six trained fieldworkers (fluent in English and Pidgin), after piloting work was carried out with residents from a local Cameroon Development Corporation community.

All completed questionnaires were downloaded using Census and Survey Processing System software (CSPro 2017) and checked for quality before being processed for analysis. Data on household characteristics, fuel use and cooking practices were summarised using descriptive statistics with appropriate hypothesis testing for continuous (*t* test/Wilcoxon) and categorical (Chi-squared test) data. To create a simple quantitative summary of the association between each household factor and use of LPG as a primary or secondary fuel (compared to exclusive solid fuel use), we used unconditional logistic regression to produce odds ratios, stratified by rural and peri-urban context. As a basic summary of the independence of household factors found to be significantly associated with LPG use, multivariable logistic regression included all factors univariately associated (*P* < 0.05 after applying a Bonferroni correction for multiple hypothesis testing) with LPG use (any and exclusive use). All analyses were conducted using Stata v14 software (StataCorp 2015).

## Results

### General Characteristics of Peri-Urban and Rural Households

A total of 1577 households completed the surveys (1334 from peri-urban Limbe (88.9% of households sampled) and 243 from rural Buea [from approximately 500 households located in the survey communities—48.6%]). The mean age of the household head was younger in peri-urban Limbe than in rural Buea (42.2 years (sd = 12.9) vs 51.8 years (sd = 16.9), respectively, *p* < 0.0005) with a lower proportion of female heads (23 vs 29%; *p* = 0.037)—Table 1. In addition, a significantly higher proportion of respondents from peri-urban households than from rural households reported having education beyond primary school level (62.7 vs 37%; *p* < 0.0005), being married (60.2 vs 46.1%; < 0.0005), having piped water (51.8 vs 30.5%; *p* < 0.0005) and having a flushing toilet (50 vs 16.1%; *p* < 0.0005). Peri-urban households reported having a higher number of residents within each house (mean = 5.1 vs 4.6; *p* = 0.005) translates into a higher number of people per room, as an indicator of crowding (mean = 2.2 vs 1.3; *p* < 0.0005).

**Table 1:** General Characteristics of Survey Populations.

Characteristic	Total Sample (*n* = 1577)		Rural Buea (*n* = 243)		Peri-urban Limbe (*n* = 1334)		*P* value
	No	%	No	%	No	%	
Head of household							
Sex							
Male	1199	76.0	172	70.8	1027	77.0	
Female	378	24.0	71	29.2	307	23.0	*0.037*
Age (Mean/sd) years	*43.7*	*14.0*	*51.8*	*16.9*	*42.2*	*12.9*	< *0.0005*
Education							
None	32	2.0	12	4.9	20	1.5	
Primary	618	39.2	141	58.0	477	35.8	
Secondary	651	41.3	64	26.3	587	44.0	
University	276	17.5	26	10.7	250	18.7	< *0.0005*
Marital status							
Married/partnership	915	58.0	112	46.1	803	60.2	
Divorced/widow(er)	133	8.4	55	22.6	78	5.9	
Single/unmarried	529	33.5	76	31.3	453	34.0	< *0.0005*
Religion							
Christian	1549	98.2	236	97.1	1313	98.4	
Other	28	1.8	7	2.9	21	1.6	0.072
Household composition							
Children							
Children < 5 years	884	56.1	108	44.4	776	58.2	0.083
Children 6–16 years	1004	63.7	131	53.9	873	65.4	*0.001*
People resident (Mean/sd)	*5.0*	*2.65*	*4.6*	*2.88*	*5.1*	*2.60*	*0.005*
Number of rooms (Mean/sd)	*2.9*	*1.55*	*4.1*	*1.65*	*2.7*	*1.43*	< *0.0005*
People per room (Mean/sd)	*2.1*	*1.23*	*1.3*	*0.97*	*2.2*	*1.22*	< *0.0005*
Water source							
Piped water	765	48.5	74	30.5	691	51.8	
Other	812	51.5	169	69.5	643	48.2	< *0.0005*
Sanitation							
Flush WC	706	44.8	39	16.1	667	50.0	
Other	871	55.2	204	83.9	407	50.0	< *0.0005*
Income and asset ownership							
Household ownership							
Owned	371	23.5	124	51.0	247	18.5	
Rented	700	44.4	27	11.1	673	50.5	
Other	506	32.1	92	37.8	414	30.9	< *0.0005*
Household income method							
Cash income only	1108	70.3	103	42.4	1005	75.3	
Other	469	29.7	140	27.6	329	24.7	< *0.0005*
Household income (CFA)							
≤ 25 k per month^a^	146	11.9	49	25.9	97	9.3	
26–50 k per month^b^	397	32.3	75	39.7	322	30.9	
51–100 k per month	388	31.5	48	25.4	340	32.6	
101 + k per month	300	24.4	17	9.0	283	27.2	< *0.0005*
Assets owned							
Electricity	1547	98.1	222	91.4	1325	99.3	< *0.0005*
Mobile phone	1532	97.2	208	85.6	1324	99.3	< *0.0005*
Television	1429	90.6	175	72.0	1254	94.0	< *0.0005*
Car	320	20.3	25	10.3	295	22.2	< *0.0005*
Motorbike	261	16.8	49	20.5	212	16.1	*0.001*

Italic values indicate statistical significance assessed at *p* < 0.05.

^a^Cut-off for ≤ 25 k is used to represent below poverty threshold (WHO—US$1.5 p/person—p/day).

^b^Cut-off for ≤ 50 k is used to represent below minimum monthly household income for Cameroon.

Respondents from peri-urban communities were more likely to rent property than those from rural communities (50.5 vs 11.1%; *p* < 0.0005). Wealth ownership also differed between the two contexts with peri-urban households being more likely to have occupations that paid a cash income (75.3 vs 42.4%; *p* < 0.0005) and reporting higher monthly household incomes above the WHO poverty threshold of 25 k CFA (90.7 vs 74.1%; *p* < 0.0005) and above the average monthly national Cameroon household income of 50 k CFA (59.8 vs 34.4%; *p* < 0.0005). In addition, peri-urban households were significantly more likely to own assets such as electricity, mobile phones, televisions and a car (*p* < 0.0005).

### Fuel Use and Cooking Patterns for Peri-Urban and Rural Households

The two most dominant primary fuel groups were wood (40.7%) and LPG (51.1%) with other fuels such as sawdust, kerosene and charcoal typically being used as secondary fuels (Table 2). All houses reporting LPG as a fuel indicated using the standard size 12.5-kg cylinder for cooking, typically obtained at the regulated refill price of 6500 CFA (USD 12), although price increases were reported in relation to transport costs and restricted supply.

**Table 2:** Primary and Secondary Fuels Use for Cooking (Including Stove and Location of Cooking), and Lighting Stratified by Rural and Peri-Urban Contexts.

Characteristic	Total sample (*n* = 1577)		Rural Buea (*n* = 243)		Peri-urban Limbe (*n* = 1334)		*p* value
	No	%	No	%	No	%	
Cooking							
Primary cooking fuel							
No cooking	8	0.5	2	0.8	6	0.5	
Electricity	4	0.3	1	0.4	3	0.2	
LPG	806	51.1	38	15.6	758	57.6	
Kerosene	45	2.9	5	2.1	40	3.0	
Charcoal	36	2.3	1	0.4	35	2.6	
Wood	641	40.7	196	80.7	445	33.4	
Sawdust	35	2.2	0	0	35	2.6	
Other	2	0.1	0	0	2	0.2	*< 0.0005*
Secondary cooking fuel							
No other fuel	373	23.9	123	51.5	250	18.9	
Electricity	7	0.5	0	0	7	0.5	
LPG	314	20.1	35	14.6	279	21.1	
Natural gas	4	0.3	2	0.8	2	0.2	
Kerosene	178	11.4	38	15.9	140	10.6	
Charcoal	273	17.5	8	3.4	265	20.0	
Wood	378	24.2	30	12.6	348	26.3	
Sawdust	32	2.1	3	1.3	29	2.2	
Other	4	0.3	0	0	4	0.3	*< 0.0005*
Exclusive usage of LPG							
All cooking with LPG	130	8.2	3	1.2	127	9.5	
LPG use (not exclusive)	986	62.5	70	28.8	916	68.7	
No LPG	461	29.2	170	70.0	291	21.8	*< 0.0005*
Location of cooking							
In house	844	53.8	54	22.4	790	59.5	
Separate building	594	37.9	79	74.3	415	31.3	
Outside	131	8.4	8	3.3	123	9.3	*< 0.0005*
Separate room for cooking	611	72.4	33	61.1	578	73.2	*< 0.0005*
Lighting							
No lighting	5	0.3	3	1.2	2	0.2	
Grid electricity	1280	81.2	197	81.1	1083	81.2	
Mini-grid electricity	266	16.9	25	10.3	241	18.1	
Solar	1	0.1	1	0.4	0	0	
Solar lantern	3	0.2	1	0.4	2	0.2	
Flashlight	3	0.2	2	0.8	1	0.1	
Kerosene lamp	15	1.0	12	4.9	3	0.2	
Candle	3	0.2	1	0.4	2	0.2	
Other	1	0.1	1	0.4	0	0	*< 0.0005*

Italic values indicate statistical significance assessed at *p* < 0.05.

In rural communities, the majority of households reported wood (mostly gathered for free) as their primary fuel (80.7% compared to 33.4% of peri-urban homes), whereas peri-urban homes were more likely to report LPG as their primary fuel (57.8 vs 15.6%; *p* < 0.0005). In peri-urban homes, kerosene (3%), charcoal (2.6%) and sawdust (2.6%) were reported by some households as a primary fuel—very few rural households reported primary use of these fuels. The majority of rural households did not use a secondary fuel at home (51.5%), whereas 81.1% of peri-urban households reported using a secondary fuel. These included a mixture of LPG (21.1%), kerosene (10.6%), charcoal (20%) and wood (26.3%) with limited use of sawdust (2.2%). To understand the extent of fuel stacking (mixed use of LPG with other fuels), exclusivity of LPG use for cooking was defined as (1) primary use of LPG with no secondary fuel or (2) LPG use reported as both primary and secondary fuels. Of the 1116 households that reported using LPG as a fuel for cooking, only 130 (11.6%) reported using it exclusively. In rural households, where only 30% reported some use of LPG as a cooking fuel, only 3 (1.2%) reported exclusive use of LPG. For peri-urban households, only 127 (9.5%) reported exclusive use of LPG with by far the majority of LPG users (916; 87.8%) ‘stacking’ fuels (using biomass and LPG). A greater proportion of peri-urban households reported cooking indoors (59.5 vs 22.4%; *p* < 0.0005) with the majority having a separate kitchen within the home (73.2%). Rural households were more likely to cook outside in a separate building used as an enclosed kitchen, located near the main house (74.3 vs 31.3%; *p* < 0.0005). The majority of households in both rural communities (91.4%) and peri-urban households (99.3%) reported use of electricity for lighting.

### Factors Associated with LPG Use in Peri-Urban and Rural Households

Associations between demographic, household and wealth characteristics with ‘any’ use of LPG for rural households (Table 3) and with ‘any’ use and ‘exclusive’ use of LPG for peri-urban households (Table 4) were assessed. For rural households, age, sex and marital status were not associated with LPG use; however, households with a head who had secondary education (OR = 3.33; 95% CI = 1.76, 6.30) and a university education (OR = 6.84; 95% CI = 2.82, 16.62) were significantly more likely to use LPG than those who had not received a secondary education. Whilst the number of people resident in the household did not affect likelihood of LPG use, access to mains water (OR = 4.04; 95% CI = 2.24, 7.28) and household sanitation (OR = 14.95; 95% CI = 6.4, 34.9) were strongly associated with an increased likelihood of using LPG. Household wealth was also strongly associated with LPG use in rural households. Households with incomes above the national average for Cameroon (50 k CFA) were significantly more likely to use LPG, than those with the highest incomes (100 + CFA) being the most likely to report using LPG (OR = 4.68; 95% CI = 1.64, 13.4). In addition, ownership of assets including a mobile phone, car and electricity were also positively associated with LPG use (*p* < 0.05). Conversely, ownership of the house was negatively associated with LPG use, owners being almost 50% less likely to report using LPG (OR = 0.51; 95% CI = 0.30, 0.91). After adjustment through multivariable analysis (Table 5), education, access to piped water, access to sanitation, ownership of a television and car ownership were found to be independently associated with any LPG use.

**Table 3:** Association of Household and Individual Characteristics with ‘Any’ LPG Use in Rural Communities.

Characteristic	‘Any’ use of LPG (*n* = 73; 30.0%)				
	No.	%	OR	95% CI	*p* value*
Head of household					
Sex					
Male	53	30.8	1.0		
Female	20	28.2	0.88	0.48, 1.62	0.683
Age					
18–35 years	17	36.2	1.0		
36–45 years	18	36.7	1.02	0.45, 2.35	0.954
46 + yrs	38	25.9	0.62	0.31, 1.24	0.174
Education					
None/primary	29	19.0	1.0		
Secondary	28	43.8	3.33	1.76, 6.30	***< 0.0005***
University	16	61.5	6.84	2.82, 16.62	***< 0.0005***
Marital status					
Single/widow(er)/divorce	43	31.6	1.0		
Married/partnership	30	30.0	0.84	0.48, 1.47	0.546
Household composition					
People per room (crowding)					
0–1.5	46	27.9	1.0		
1.6–2.0	17	37.8	1.57	0.79, 3.14	0.201
2.1–9.0	10	30.3	1.12	0.50, 2.55	0.778
Water source					
No piped water	35	20.7	1.0		
Piped water	38	51.4	4.04	2.24, 7.28	***< 0.0005***
Sanitation					
No flush WC	42	20.6	1.0		
Flush WC	31	79.5	14.95	6.40, 34.9	***< 0.0005***
Income and asset ownership					
Household ownership					
Doesn’t own house	44	37.0	1.0		
Own house	29	23.4	0.52	0.30, 0.91	0.022
Household income method					
No paid exclusively in cash	36	25.7	1.0		
Cash income only	37	35.9	1.62	0.93, 2.82	0.087
Household income (CFA)					
< 50 k per month^a^	29	23.4	1.0		
50–100 k per month	21	43.8	2.55	1.26, 5.16	0.009
101 + k per month	10	58.8	4.68	1.64, 13.39	0.004
Assets owned					
Electricity	71	32.0	4.47	1.01, 19.70	0.048
Mobile phone	72	34.6	18.0	2.41, 134.2	0.005
Television	68	38.9	8.01	3.07, 20.91	***< 0.0005***
Car	19	76.0	9.80	3.72, 25.81	***< 0.0005***
Motorbike	14	30.4	1.02	0.51, 2.06	0.943

*P values in bold/italics are statistically significant at *p* < 0.05 after Bonferroni correction has been applied.

^a^Cut-off for < 50 k is used to represent below minimum monthly household income for Cameroon.

**Table 4:** Association of Household and Individual Characteristics with ‘Any’ and ‘Exclusive’ LPG Use in Peri-Urban Communities.

Characteristic	‘Any’ use of LPG (*n* = 1043)					‘Exclusive’ use of LPG (*n* = 127)				
	No.	%	OR	95% CI	*p* value*	No.	%	OR	95% CI	*p* value*
Head of household										
Sex										
Male	807	78.6	1.0			98	9.5	1.0		
Female	236	76.9	0.91	0.67, 1.23	0.526	29	9.5	0.99	0.64, 1.53	0.960
Age										
18–35 years	401	84.1	1.0			91	19.1	1.0		
36–45 years	346	81.0	0.81	0.57, 1.14	0.229	24	5.6	0.28	0.16, 0.40	***< 0.0005***
46 + years	296	68.8	0.42	0.30, 0.58	***< 0.0005***	12	2.8	0.12	0.07, 0.23	***< 0.0005***
Education										
None/primary	312	62.8	1.0			19	3.8	1.0		
Secondary	496	84.5	3.23	2.42, 4.31	***< 0.0005***	71	12.1	3.46	2.06, 5.83	***< 0.0005***
University	235	94.0	9.29	5.34, 16.2	***< 0.0005***	37	14.8	4.37	2.46, 7.78	***< 0.0005***
Marital status										
Single/widow(er)/divorce	442	76.9	1.0			76	13.2	1.0		
Married/partnership	601	79.2	1.14	0.88, 1.49	0.311	51	6.7	0.47	0.33, 0.69	***< 0.0005***
Household composition:										
People per room (crowding)										
0–1.5	382	82.5	1.0			77	16.6	1.0		
1.6–2.0	271	82.1	0.97	0.88, 1.49	0.889	28	8.5	0.46	0.29, 0.73	***< 0.0005***
2.1–9.0	390	72.1	0.55	0.40, 0.74	***< 0.0005***	22	4.1	0.21	0.13, 0.35	***< 0.0005***
Water source										
No piped water	430	66.9	1.0			50	7.8	1.0		
Piped water	613	88.7	3.89	2.92, 5.19	***< 0.0005***	77	11.4	1.49	1.02, 2.16	0.037
Sanitation										
No flush WC	434	65.1	1.0			52	7.8	1.0		
Flush WC	609	91.3	5.64	4.12, 7.71	***< 0.0005***	75	11.2	1.50	1.03, 2.17	0.033
Income and asset ownership										
Household ownership										
Don’t own house	859	79.0	1.0			117	10.8	1.0		
Own house	184	74.5	0.78	0.56, 1.07	0.120	10	4.1	0.35	0.18, 0.68	***0.002***
Household income method										
Not all cash income	223	67.8	1.0			18	5.5	1.0		
Cash income only	820	81.6	2.11	1.59, 2.79	***< 0.0005***	109	10.9	2.10	1.26, 3.52	***< 0.0005***
Household income (CFA)										
≤ 50 k per month^a^	283	67.5	1.0			38	9.1	1.0		
51–100 k per month	274	80.6	2.00	1.42, 2.80	***< 0.0005***	30	8.8	0.97	0.59, 1.60	0.906
101 + k per month	265	93.6	7.07	4.21, 11.9	***< 0.0005***	36	12.7	1.46	0.90, 2.37	0.124
Assets owned										
Electricity	1039	78.4	4.54	1.21, 17.0	0.025	127	9.6	---^b^	---	---
Mobile phone	1040	78.6	8.54	2.20, 33.3	***0.002***	126	9.5	0.95	0.12, 7.53	0.959
Television	1018	81.2	9.49	5.79, 15.5	***< 0.0005***	117	9.3	0.72	0.36, 1.43	0.351
Car	268	90.9	3.39	2.23, 5.16	***< 0.0005***	35	11.9	1.38	0.92, 2.09	0.124
Motorbike	161	79.3	1.09	0.75, 1.57	0.648	19	9.4	0.96	0.58, 1.61	0.888

*P values in bold/italics are statistically significant at *p* < 0.05 after Bonferroni correction has been applied.

^a^Cut-off for < 50 k is used to represent below minimum monthly household income for Cameroon.

^b^Estimate not possible (127/129 using electricity).

**Table 5:** Independent Association Between Household and Individual Characteristics with ‘Any’ LPG Use in Rural Communities*.

Characteristic	‘Any’ use of LPG (*n* = 1043)		
	OR	95% CI	*p* value
Head of household			
Education			
None/primary	1.0		
Secondary	2.49	1.89, 3.28	< 0.0005
University	3.81	2.35, 6.17	< 0.0005
Household composition			
Water source			
No piped water	1.0		
Piped water	1.56	1.16, 2.10	0.003
Sanitation			
No flush WC	1.0		
Flush WC	3.91	2.80, 5.45	< 0.0005
Income and asset ownership			
Assets owned			
Television	7.07	4.46, 11.22	< 0.0005
Car	1.92	1.26, 2.90	0.002

*Adjustment for all factors univariately associated with ‘any’ and ‘exclusive’ use of LPG.

For the peri-urban setting, the household heads of an older age (46+ years) were significantly less likely to report using any LPG than younger ages (18–35 years)—OR = 0.42; 95% CI = 0.38, 0.58 (Table 4)—the association was more pronounced for exclusive use of LPG (OR = 0.12; 95% CI = 0.07, 0.23). Although not associated with any use of LPG, respondents who indicated being married or in a partnership were significantly less likely to report exclusive use of LPG (OR = 0.47; 95% CI = 0.33, 0.69). As with the rural setting, increasing level of education and having access to mains water and household sanitation were also significantly associated with the likelihood of using LPG and also, for the peri-urban community, with exclusive use of LPG (*p* < 0.05, after Bonferroni correction). Households identified as having the highest person-to-room ratio (crowding indicator) were significantly less likely to report using any LPG (OR = 0.55; 95% CI = 0.40, 0.74) and exclusive use of LPG (OR = 0.21, 0.13, 0.35). Whilst, similar to rural households, indicators of household wealth (being paid exclusively in cash, higher income bracket and ownership of a mobile phone, a car and a television) were significantly associated with any LPG use (*p* < 0.05, after Bonferroni correction). Only the method of receiving income (in cash rather than kind) was associated with an increased likelihood of exclusive use (OR = 2.10; 95% CI = 1.26, 3.52).

Multivariable analysis of peri-urban homes identified a number of factors independently associated with both ‘any’ and ‘exclusive use’ of LPG use (Table 6). Increasing level of education, younger age, access to piped water and household sanitation, payment in cash rather than kind, a higher level of income and ownership of a television were all significantly (*p* < 0.05) and independently associated with any LPG use. For exclusive use of LPG, increasing education, younger age, being single, having a less crowded household and being paid in cash all demonstrated significant independent associations.

**Table 6:** Independent Associations Between Household and Individual Characteristics with ‘Any’ and ‘Exclusive’ LPG Use in Peri-Urban Communities*.

Characteristic	‘Any’ use of LPG (*n* = 1043)			‘Exclusive’ use of LPG (*n* = 127)		
	OR	95% CI	*p* value	OR	95% CI	*p* value
Head of household						
Age						
18–35 years	1.0			1.0		
36–45 years	0.66	0.41, 1.04	0.075	0.28	0.17, 0.46	< 0.0005
46 + years	0.31	0.20, 0.48	< 0.0005	0.11	0.06, 0.21	< 0.0005
Education						
None/primary	1.0			1.0		
Secondary	1.82	1.26, 2.65	0.002	2.42	1.40, 4.19	0.002
University	3.39	1.59, 7.21	0.002	3.20	1.17, 5.91	< 0.0005
Marital status						
Single/divorce	xxx			1.0		
Married/partner	xxx			0.45	0.30, 0.68	< 0.0005
Household composition						
People per room (crowding)						
0–1.5	1.0			1.0		
1.6–2.0	1.12	0.68, 1.84	0.656	0.45	0.28, 0.74	0.001
2.1–9.0	0.78	0.51, 1.20	0.260	0.22	0.13, 0.37	< 0.0005
Water source						
No piped water	1.0			xxx		
Piped water	1.90	1.26, 2.85	0.002	xxx		
Sanitation						
No flush WC	1.0			xxx		
Flush WC	2.22	1.45, 3.40	< 0.0005	xxx		
Income and asset ownership						
Household income method						
Not all cash income	1.0			1.0		
Cash income only	1.54	1.04, 2.29	0.033	1.83	1.05, 3.20	0.034
Household income (CFA)						
≤ 50 k per month^1^	1.0			xxx		
51–100 k per month	1.53	1.04, 2.25	0.031	xxx		
101 + k per month	2.73	1.51, 4.93	0.001	xxx		
Assets owned						
Mobile phone	1.47	0.19, 11.13	0.711	xxx		
Television	6.97	3.52, 13.77	< 0.0005			
Car	1.50	0.89, 2.53	0.130			

*Adjustment for all factors univariately associated with ‘any’ and ‘exclusive’ use of LPG.

## Discussion

This study has summarised both patterns of fuel use in rural and peri-urban communities in south-west Cameroon and individual and household characteristics associated with using LPG as a primary and exclusive fuel.

### Fuel Use Patterns and ‘Stacking’

For the LACE studies, regions were chosen from Anglophone south-west Cameroon to identify peri-urban and rural communities with some market penetration of LPG, where market expansion is planned in the near future. A comparison of national data for education, access to piped water and income (Table 7) identifies that levels of poverty in the LACE study sample are lower than seen nationally, but education and access to clean water in the home are similar to the country as a whole.

**Table 7:** Comparison of Data on Education, Water Supply, Primary Cooking Fuel and Poverty for the LACE Census Sample and Nationally for Cameroon.

Characteristic	LACE census survey findings			National survey findings	
	All (%)	Rural (%)	Peri-urban (%)	Value (%)	Source
At least primary education (%)	98	58.0	98.5	75	MDG (2015) Report (NIS, 2015)
Piped water supply to homes^a^	48.5	30.5	51.8	45	2007 data in (MINPROFF 2012)
Income (poverty)^b^	11.9	25.9	9.3	37.5	ECAM (2014) survey (NIS 2014)

^a^For National data, the description is of clean (drinkable) water in the house.

^b^Defined as less than 913 CFA/day (about US$1.5) for LACE and ECAM.

The LACE surveys identified primary use of solid fuel for cooking (almost exclusively wood) at similar levels to those reported nationally (NIS, 2015) in both the rural communities (LACE 81.1% vs National 87.5%) and peri-urban communities (LACE 38.6% vs National 36.8%). In terms of using LPG for cooking, 57.6% of peri-urban homes and 15.6% of rural homes reported LPG as their primary fuel. In relation to the government stated national aspirational target of 58% use by 2030, an estimated 37% of homes currently use LPG as their primary cooking fuel (based on 45% of the Cameroon population being rural) (Worldbank 2016). Such extrapolation should be treated with caution as the LACE study population is not representative of the national population. Geographically, the communities were located in an Anglophone costal region with a relatively well-established LPG market and infrastructure. In addition, when compared with national data (NIS 2014), the LACE population had higher reported levels of education (at least primary school education: LACE 98% vs National 75%) and lower levels of poverty (income less than 913 CFA/day (about US$1.5): LACE 11.9% vs National 37.5%). Despite these differences which favour household use of LPG, current primary use of LPG is well below the government target.

One recognised policy issue in supporting communities to switch to cleaner fuels/technologies is the problem of ‘fuel/stove stacking’ whereby households that use cleaner cooking fuels/technologies do so alongside traditional polluting fuels/technologies (Dickinson et al. 2016). In the LACE peri-urban area, only 9.5% of the 70% of LPG users indicated doing so exclusively, and in the rural area, only 1.2% reported exclusive use of LPG (70% indicating no use of LPG). The issue of stove stacking is not new; over the last 20 years, empirical evidence exists from LMICs that households gaining access to LPG were only marginally displacing traditional fuels (Masera et al. 2000; Masera and Navia 1997). More recently, large-scale programmes including the substitution of household kerosene with LPG in Indonesia (Andadari et al. 2014) and rural electrification in China (Trac 2011) have stressed the problem of stacking. Use of multiple fuels is a barrier to achieve reductions in household air pollution necessary to achieve WHO indoor air quality targets to positively impact health which require almost exclusive use of clean fuels or technologies (Johnson and Chiang 2015). Addressing the problem of fuel stacking is not straightforward, and it is necessary to understand ‘the dynamic interplay among household behaviour, culture, environment, energy and technology’ to identify how best to support more exclusive use of cleaner fuels and technologies (Ruiz-Mercado and Masera 2015).

### Factors Influencing Use of LPG as a Clean Fuel

A range of households and individual characteristics were found to be associated with LPG use. In both rural and peri-urban homes, level of education was independently associated with use of LPG with a ‘dose–response’ relationship between increasing level of education and an increasing likelihood to use any LPG (rural and peri-urban) and an exclusive LPG (peri-urban). The importance of education in the transition to modern and cleaner cooking fuels is well documented (Makonese et al. 2017; Mekonnen and Kohlin 2008; Nlom and Karimov 2015). In their analysis of determinants of household cooking fuels across Southern Africa, Makonese et al. (2017) found education to be an important predictor of type of primary cooking fuel in Lesotho, Namibia, Swaziland, Zambia and Zimbabwe. Education is important in promoting the benefits of clean energy, clean fuels and the health implications of using traditional fuels for cooking, and the authors suggest that better education for household heads, promotion of efforts to specifically target less educated populations and tailoring educational materials appropriately would create a shift reducing the chances of choosing traditional fuels over modern cleaner options such as LPG and electricity.

The evidence for the role of age of household head and the main cook on adoption of cleaner cooking technologies and fuels has been inconclusive (Lewis and Pattanayak EHP 2012). A systematic review identified 29 studies with 38% identifying a significant association between use of cleaner cooking technologies/fuels and increasing age and 24% finding a significant association with younger ages (Lewis and Pattananyak EHP 2012). We observed a strong association between younger age of the cook and primary use of LPG fuel, likely reflecting a greater willingness to accept more modern technologies and an aspirational value attached to LPG fuel.

Another well-documented determinant of uptake of cleaner modern cooking fuels and technologies is household wealth. In this analysis, a number of indicators of wealth (including household income) were found to be significantly associated with LPG use. In the rural community access to piped water and household sanitation, ownership of a range of assets, a higher household income and being paid exclusively in cash were all positively associated with reported use of LPG—although only household sanitation access was independently associated in the multivariable analysis. For the peri-urban community, access to sanitation, a higher household income, being paid exclusively in cash and asset ownership of a television were all independently associated with LPG use (although only being paid exclusively in cash as an indicator of wealth was independently associated with exclusive use). Wealth and income have been identified as among the most important predictors of use of clean fuels for cooking in Southern Africa (Makonese et al. 2017), Cameroon (Nlom and Karimov 2015), Nigeria (Desalu et al. 2012) and Ethiopia (Gebreegziabher et al. 2012). Whilst availability of finance is clearly related to the ability to purchase the equipment needed to adopt LPG as a clean domestic fuel, it is also important in sustaining its use through the purchase of repeat refills to meet routine cooking requirements. In a mixed method, systematic review of barriers and enablers to adoption and sustained use of LPG and other clean fuels, Puzzolo and colleagues highlighted the initial cost of LPG (stove and fuel) as the most frequently reported barrier to adoption by people with limited resources and was a particular problem in areas where biomass could be collected as a fuel for free (Puzzolo et al. 2016). The authors also observed that the ongoing costs of using cleaner fuels were a reported barrier to both its sustained use and the extent to which it was used exclusively, displacing use of traditional polluting biomass. Conversely, for communities where traditional fuels such as wood and charcoal were purchased, the relative cost of LPG was reported to be less of a barrier due to cost savings from reducing purchasing of these fuels.

Despite identifying these key household income and socio-economic factors associated with both ‘any’ and ‘exclusive’ use of LPG, caution must be taken regarding their interpretation as drivers for transition to cleaner energy in relation to policy. Such a simplistic interpretation overlooks the role of human dimensions in choosing domestic fuels and ignores the complexity of the dynamic interactions between preferences, cultural norms, habits and behaviours in adopting and using clean fuels (Ruiz-Mercado and Masera 2015). In addition, there are many factors outside those at a household level that are important in both the adoption and sustained use of cleaner modern fuels. Puzzolo et al. (2016) identified seven domains relevant to the adoption and use of household energy, only two of which primarily relate to households and communities (Box 1).

**Box 1:** Seven domains relevant to adoption and sustained use of household energy (Source: Puzzolo et al. 2016).

1: Fuel and technology characteristics
2: Household and setting characteristics
3: Knowledge and perceptions
4: Financial, tax and subsidy aspects
5: Market development
6: Regulation, legislation and standards
7: Programme and policy mechanisms

The authors identified that, whilst some factors, such as income, meeting cooking needs, fuel availability were of particular importance, none were sufficient on their own to influence adoption and sustained use of clean fuel. In addition, whilst some factors related to circumstances and perspectives at the household and local community, others related to wider programmatic and societal issues. Therefore, it is important that factors from all seven domains receive attention in the planning, implementation and evaluation of initiatives to introduce and scale up clean fuels (Puzzolo et al. 2016).

## Conclusion

This cross-sectional survey of households in rural and peri-urban communities of south-west Cameroon has identified current cooking fuel use practices and associated factors in a region with a relatively well-established LPG market, lower levels of poverty and national Cameroon picture. The proportion of households using LPG as a primary cooking fuel is well below the national target set by the Cameroon government (58% by 2030). In addition, the problem of fuel stacking is clear with less than 10% of LPG using households in urban areas reporting they do so exclusively (only 1% of rural households). Household-level factors identified to be associated with adoption and sustained/exclusive use of LPG, including household wealth and level of education, have been well documented as both important potential barriers (lower levels) and enablers (higher levels) in using cleaner fuels and technologies. However, to develop programmes and policies to support their scaled transition, switching from polluting traditional fuels, it is necessary to consider factors across the whole LPG ecosystem. With assistance from the Global LPG Partnership, the Cameroon government launched an LPG Masterplan in 2016 to help achieve its stated aspirational LPG adoption goal (GLPGP 2016). The LPG Masterplan sets out important recommendations around investment, infrastructure, market development and regulation necessary to ensure an LPG market that meets the conditions for effective sustained expansion. The LACE studies have been conducted in conjunction with publication of the LPG Masterplan to help identify how to support households, make the transition to LPG as a clean fuel to benefit the health, environments and lives of their communities.
